# Using cell-free DNA for HCC surveillance and prognosis

**DOI:** 10.1016/j.jhepr.2021.100304

**Published:** 2021-05-10

**Authors:** Nguyen H. Tran, John Kisiel, Lewis R. Roberts

**Affiliations:** 1 Department of Oncology, Mayo Clinic, Rochester, Minnesota, United States; 2 Division of Gastroenterology and Hepatology, Mayo Clinic, Rochester, Minnesota, United States

**Keywords:** cfDNA, ctDNA, circulating biomarker, liver cancer, hepatocellular carcinoma, cell-free nucleic acids, AFP, alpha-fetoprotein, cfDNA, circulating cell-free DNA, CNV, copy number variants, ctDNA, circulating tumour DNA, HCC, hepatocellular carcinoma, NGS, next-generation sequencing, SNV, single nucleotide variants, WGS, whole genome sequencing

## Abstract

Hepatocellular carcinoma (HCC) is the most common form of primary liver cancer. Its incidence is rising faster than any other cancer in the United States and it remains one of the leading causes of cancer-related deaths worldwide. While advances in massive parallel sequencing and integration of ‘omics information have transformed the field of oncology, tissue access is often limited in HCC and a single biopsy is poorly representative of the known genetic heterogeneity of tumours. Liquid biopsy has emerged as a promising strategy for analysing circulating tumour components including circulating tumour DNA. Cell-free DNA and tumour DNA are derived from necrotic, apoptotic and living eukaryotic cells. The profiling of genetic and epigenetic alterations in circulating cell-free DNA has potential clinical applications including early disease detection, prediction of treatment response and prognostication in real time. Novel biomarker candidates for disease detection and monitoring are under study. Of these, methylation analyses of circulating tumour DNA have shown promising performance for early HCC detection in at-risk patients. Assessments of assay performance in longitudinal validation cohorts are ongoing. Implementation of liquid biopsy for HCC will likely improve upon the current surveillance strategy. This review summarises the most recent developments on the role and utility of circulating cell-free DNA in the detection and management of HCC.

**Key points**

• Early detection
  ○ Early detection of hepatocellular carcinoma leads to early curative treatment and improves survival.
  ○ Several methylation panels assayed from plasma DNA have demonstrated high sensitivity and specificity in detecting early disease in at-risk individuals.
• Potential clinical utility of cell-free DNA
  ○ Longitudinal prospective studies are ongoing.
  ○ Detect cancer in individuals at high risk.
  ○ Measure residual disease following surgery, ablation or transplant with risk stratification for adjuvant therapy.
  ○ Enable treatment selection.
  ○ Elucidate mechanisms of resistance and disease progression.

## Introduction

Hepatocellular carcinoma (HCC), the most common form of primary liver cancer, is one of the leading causes of cancer-related deaths worldwide.^1^ Each year, more than 800,000 individuals are diagnosed globally. The incidence of HCC is rising faster than that of any other cancer in the United States,^2^ driven by HCV-associated cirrhosis and the rising prevalence of non-alcoholic fatty liver disease.^3^ The annual risk of developing HCC in high risk groups, including chronic carriers of HBV and patients with cirrhosis (of infectious, metabolic or alcoholic aetiology) is 2–4% per year.^4^ Several studies have demonstrated that HCC surveillance is associated with early detection, receipt of curative treatment, and improved survival.^5^^,^^6^ Consequently, for those patients at high risk, clinical practice guidelines recommend biannual HCC surveillance by ultrasound imaging with or without serum alpha-fetoprotein (AFP) testing.^7^^,^^8^ However, several limitations exist with this approach; the aggregate sensitivity is low at 63%, especially for those with early disease within and outside of Milan criteria for liver transplantation, and surveillance is underutilised.^9^ A recent meta-analysis involving more than 118,000 patients showed a pooled estimate for surveillance use of 24% (95% CI 18.4–30.1), with variable usage depending on the level of care (subspecialty care or primary care).^10^ Furthermore, the diagnosis of HCC is made using radiological or histological criteria. Liver biopsy is invasive, poorly reflects the temporal and spatial heterogeneity within the tumour, and is often not available. This presents a large window of opportunity for the development of novel biomarkers that can detect HCC early and can accurately predict outcomes.

Currently, liver resection and local ablation remain the mainstays of curative therapy, as liver transplant is limited by the size of the donor pool and stringent eligibility criteria.^11^^,^^12^ After resection, recurrence within the residual cirrhotic liver is high, with more than 50% of patients developing HCC recurrence within 3 years and 70–80% recurring within 5 years; the 5-year survival rate for these patients is approximately 40–50%.[13], [14], [15], [16], [17] Over the last few years, significant advances have been made in the management of patients with advanced HCC. Since 2007, sorafenib^18^ – an oral multikinase inhibitor – and subsequently lenvatinib,^19^ have been the first-line systemic therapies for patients with advanced HCC. Recently, atezolizumab – an anti-PD-L1 antibody – in combination with bevacizumab – a monoclonal anti-VEGF antibody – showed superior outcomes (67.2% overall survival (OS) at 12 months *vs.* 54.6% with sorafenib),^20^ which led to FDA approval of this combination as first-line systemic treatment for advanced HCC. Second-line treatments include the multikinase inhibitors cabozantinib^21^ and regorafenib,^22^ the anti-VEGF antibody ramucirumab,^23^ the anti-PD1 antibodies nivolumab,^24^ pembrolizumab^25^ and the combination of nivolumab with the CTLA-4 inhibitor, ipilimumab.^26^

With the exception of ramucirumab, for which AFP >400 ng/dl is associated with response, there are no biomarkers to stratify patients with HCC. Thus, emerging novel biomarkers such as circulating tumour DNA (ctDNA) – the tumour-specific component of circulating cell-free DNA (cfDNA) – have garnered substantial attention in the last few years, owing to their potential to address 3 key clinical problems. First, can they be used to detect early HCC at a stage when current surveillance modalities are insensitive and difficult to access? Second, will cfDNA levels obtained before or after surgery be prognostic of long-term outcomes? Lastly, can cfDNA associated with HCC predict response to treatment? In this review, we discuss the most recent developments on the role and utility of circulating cfDNA in the detection and prognostication of HCC.

## Liquid biopsy in HCC

### Role of cfDNA

**Fig. 1:**
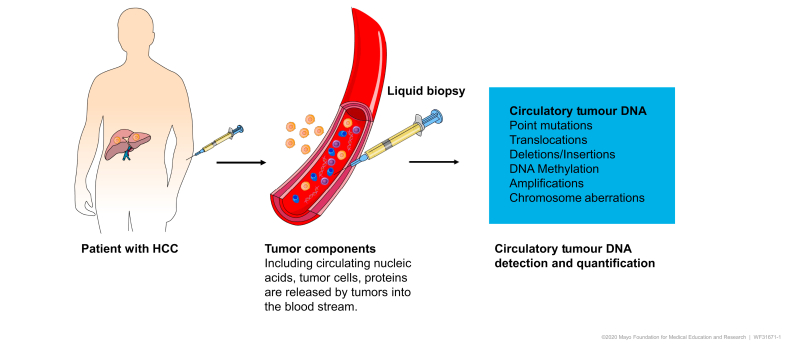
Detection and quantification of circulating tumour DNA. Tumour components including circulatory nucleic acids, tumour cells and proteins are released into the blood stream. Of these, circulating tumour DNA analysis provides information regarding mutations, translocations, deletions/insertions, amplifications and DNA methylation patterns.

Liquid biopsy, the minimally invasive assay of circulating cancer-associated biomarkers such as circulating nucleic acids, circulating tumour cells, miRNAs and exosomes, has several potential clinical applications.^27^^,^^28^ Of these, the analysis of cfDNA is currently the most promising in HCC. Circulating cfDNA refers to fragments of DNA detected in both healthy individuals and patients with cancer.^29^^,^^30^ cfDNA mostly comprises DNA shed from the normal turnover of lymphoid and myeloid cells,^31^ with ctDNA making up less than 1% of total cfDNA in patients with cancer (Fig. 1).^32^ Most circulating cfDNA fragments are double-stranded, exist in plasma or serum, and are longer than 167 base pairs.[33], [34], [35] This size approximates the length of DNA wrapped around a single nucleosome, which may protect DNA from degradation by blood nucleases. In contrast, ctDNA fragments, which are released by necrotic or apoptotic tumour cells, are typically shorter than 150 base pairs; these size differences, as well as sequence variation or epigenetic modifications, may be exploited to identify tumour-specific sequences.^34^

Indeed, cancer-specific alterations in ctDNA are measurable by next-generation sequencing (NGS) and targeted PCR-based technologies.^36^ Early studies demonstrated that ctDNA harbour molecular characteristics known to be present in the genomic DNA of cancers, such as methylation changes[37], [38], [39], [40] and point mutations,[41], [42], [43], [44] which reflect the molecular heterogeneity of a cancer that may be comprised of different tumour clones and metastases. This non-invasive approach involving cfDNA/ctDNA sampling in liquid biopsy is of great interest as it overcomes the limitation of traditional tissue biopsy and temporally reflects the clonal evolution in real time. Potential clinical utilities of cfDNA/ctDNA have been and are being investigated for the detection of HCC,[45], [46], [47], [48] disease monitoring,[49], [50], [51] and prognostication.^41^^,^^43^^,^^52^

### Storage and detection technology platforms

CtDNA targets must be detected among the background of total cfDNA. Given the short half-life (of between 16 mins and 2.5 h^53^) and low abundance of ctDNA, it is important to select the right sample collection tube and optimal processing methods to ensure successful DNA isolation. The concentration of cfDNA has been shown to be about 20-fold higher in serum than in matched plasma samples, predominantly as a result of the clotting process in the collection tube.^54^ Thus, plasma is the preferred biological sample. During handling and processing of whole blood into plasma, lysis of leukocytes can cause enormous contamination of plasma with genomic DNA, reducing if not eliminating, resolution for ctDNA targets. CtDNA is most commonly extracted from peripheral blood plasma; in contrast, processing of whole blood to serum results in shearing and lysis of leukocytes in the clot matrix. DNAases in circulation or sampled whole blood can cause potential loss of global cfDNA from the time of collection to processing to storage to analysis. Specialised tubes such as LBguard (Biomatrica, San Diego CA), Streck (La Vista NE) or Cell-Free DNA Collection (Roche, Basel Switzerland) tubes contain proprietary cfDNA preservation and cell stabilisation buffers for better ctDNA yield and quality.^55^ The separation of plasma from whole blood requires a 2000 x g centrifugation. The recommended storage temperature is -80^o^C.

In the last decade, more robust methods with high analytical sensitivity have been developed for ctDNA analysis. These include digital droplet PCR, BEAMing (Beads, Emulsion, Amplification, Magnetic) technology, quantitative allele-specific real-time target and signal amplification and the resulting TELQAS (target enrichment long-probe quantitative amplified signal).[56], [57], [58] These methods allow for more targeted analyses of single nucleotide mutations or methylation changes for example. While the targeted PCR approach is lower cost and has very high sensitivity (mutation to wild-type ratios as low as 0.01%),^59^ small gene panels will miss mutations that are not selected. Much more costly, untargeted NGS approaches include whole genome sequencing (WGS), whole exome sequencing (WES) and whole genome bisulfite sequencing to screen the genome, exome or methylome for the discovery of known and new aberrations.^32^^,^^60^ A hybrid of these approaches uses NGS after targeted capture of hundreds or even thousands of known allelic or methylated variants.

## Early detection of HCC

Early detection of HCC is critical as curative approaches are available when the tumour is small. Despite the current recommendations, standard ultrasound has several disadvantages including suboptimal performance (with sensitivity as low as 42% for lesions smaller than 1 cm), being operator dependent, and involving a cumbersome process for patients. Both CT and MRI perform better for the early detection of HCC. A systematic review including 20 studies reported a pooled sensitivity of 67.5% at 92.5% specificity for CT and 80.6% at 84.8% specificity for MRI.^61^ For lesions greater than 2 cm, these imaging modalities showed great sensitivity and specificity. However, for small tumours and those that lack arterial-phase hyper-enhancement, which may be up to 40% of HCC, these imaging modalities are limiting in diagnosing HCC. For HCC ≤1 cm in size, detection rates can be as low as 34% and 10% for MRI and CT, respectively.^62^ Other disadvantages of these tests include cost, access and radiation exposure. Thus, there is a clear need for non-invasive biomarkers that can identify early disease, ideally those patients with lesions <2 cm, and thereby minimise morbidity and mortality associated with late-stage disease. Such measures would also enable the implementation of more efficient and cost-effective surveillance strategies.

### Quantitative cfDNA measurement

Early studies investigated the clinical utility of cfDNA concentration as a biomarker for detecting HCC. For example, Iizuka *et al.* observed an increase in cfDNA concentration in 52 patients with HCV-associated HCC compared to HCV carriers, with an optimal cut-off of 73.0 ng/ml.^63^ Since then, several studies have reported significantly higher cfDNA concentrations in patients with HCC compared to those with chronic hepatitis and almost 20 times that of healthy controls.[64], [65], [66] Despite these findings, several weaknesses exist with this approach. These studies were carried out in both serum and plasma samples, reflecting the different concentrations of cfDNA. Furthermore, different studies utilised different cut-off values to discriminate high or low cfDNA concentration, suggesting that the level is assay platform dependent. Importantly, quantitative analysis of cfDNA does not provide information about the origin of the tumour, molecular alterations or potential targets.

Recently, a model integrating cfDNA levels with age and AFP reported higher detection capability, with an AUC of 0.98 (95% CI 0.92–1.00) at sensitivity of 87% and specificity of 100%.^67^ This suggests that quantitative cfDNA analysis may still hold promise when it is combined with other protein or genetic markers for the detection of HCC.

### Qualitative cfDNA measurement

Significant research interest in recent years has focused on the molecular characteristics of ctDNA in plasma, including methylation patterns and hotspot mutations. In general, these studies have yielded great detection potential in HCC. Table 1 summarises some of the most recent results and performance of these biomarkers.

**Table 1:** **Performance of ctDNA for early detection of HCC in selected studies**.

Study	Target	Patients	Sensitivity	Specificity	AUC	Limitations
**ctDNA methylation profiling**						
Wang *et al.*,^81^ 2020	21 DMRs	148 HCC, 84 healthy controls (training) 112 HCC, 96 healthy controls (validation)	82.9%	94%	0.94	Healthy controls
Yang *et al.*,^83^ 2020	39 DMRs	140 HCC, 84 healthy controls (diagnostic) 155 HCC, 96 healthy controls, 21 HBV, 34 benign liver disease (validation)	81% (diagnostic) 75% (validation)	91% (diagnostic) Validation not reported	0.93 (diagnostic) 0.90 (validation)	Low number of at-risk controls
Chalasani *et al.*,^110^ 2020	*HOXA1, EMX1, TSPYL5, B3GALT6*, AFP, AFP-L3, and sex	135 HCC, 302 controls (viral and non-viral) BCLC 0-A: 56%	71% (early stage) 81% (pooled)	89%	0.86 (early stage) 0.91 (pooled)	Follow-up study of Kisiel *et al.*
Roy *et al.**^86^**,* 2019	Not reported	60 HCC, 10 benign liver disease, 30 healthy, 30 other cancer type	95%	98%	Not reported	Follow-up study of Xu *et al.* Small cirrhotic controls, different stages of cancer
Kisiel *et al.**^80^**,* 2019	6 markers	95 HCC, 51 cirrhosis, 98 healthy controls BCLC 0-A: 48%	95% (91% for BCLC 0/A)	92%	0.94	Small cirrhotic controls, small number with early-stage disease
Cai *et al.*,^84^ 2019	5-hmC based 32 gene panel	335 HCC, 263 HBV/cirrhosis, 522 healthy controls (training) BCLC 0-A: 100% 809 HCC, 129 HBV/cirrhosis, 256 healthy controls (validation) BCLC 0-A: 27%	89.6% (training) 82.7% (validation)	78.9% (training) 76.4% (validation)	0.92 (training) 0.88 (validation)	
Oussalah *et al.**^75^**,* 2018	*SEPT9*	98 HCC, 191 cirrhosis BCLC 0-A: 25%	81%-97%	69%-96%	0.94 (pooled)	Single target
Xu *et al.,**^82^* 2017	10 markers	1098 HCC, 835 healthy controls Stage I: 16%	83%-86%	91%-95%	0.94-0.97	Healthy controls
**ctDNA mutation profiling**						
Tao *et al.*,^111^ 2020	Somatic copy number aberrations	108 HCC, 101 HBV controls (discovery) BCLC 0-A: 67% 89 HCC, 86 HBV (validation) BCLC 0-A: 100%	70% (early stage)	95%	0.89 (pooled)	Limited to HBV controls, retrospective data
Qu *et al.*,^94^ 2019	4 genes (*TP53, CTNNB1, AXIN1, TERT*) + HBV insertion site, AFP, DCP	65 HCC, 70 non-HCC (training) 331 at-risk patients (validation)	85% (training) 100% (validation)	93% (training) 94% (validation)	Not reported	Low positive predictive value of 17%, younger healthy control in training cohort
Cai *et al.*,^91^ 2019	Copy number variants and single nucleotide variants, AFP, AFP-L3, DCP	34 resected HCC	100%	Not reported	Not reported	Small sample size

AFP, alpha-fetoprotein; BCLC, Barcelona Clinic liver cancer; ctDNA, circulating tumour DNA; DMRs, differentially methylated regions; DCP, des-γ-carboxy-prothrombin; HCC, hepatocellular carcinoma.

### Analysis of epigenetic changes in ctDNA in HCC

Epigenetic modification, such as DNA methylation, plays a crucial role in regulating gene activity both in normal and cancerous cells.^68^ While cancer cells exhibit global loss of DNA methylation, hypermethylation at CpG islands and promoters is highly tumour specific and quantifiable.[69], [70], [71], [72] In HCC specifically, inactivation of tumour suppressor genes by aberrant methylation of CpG islands is thought to play an early and important role in the pathogenesis of disease.^73^^,^^74^ Hence, screening for these changes that are highly unique to the tumours may allow for early cancer detection. Indeed, recent studies have identified several diagnostic methylation markers that can discriminate HCC from controls with excellent sensitivity and specificity (Table 1). In 1 study, Oussalal and colleagues identified single-target *SEPT9* as a good diagnostic cfDNA methylation marker.^75^ Among 98 patients with HCC and 191 controls, methylated *SEPT9* in plasma DNA yielded high accuracy for the detection of HCC with an AUC of 0.94. This test has received the CE Mark for the detection of liver cancer among patients with cirrhosis in Europe (October 2018).^76^ A prospective multicentre study to determine its diagnostic performance in a US cohort had completed recruitment as of January 2020 (ClinicalTrials.gov Identifier: NCT03804593) and a larger phase II prospective cross-sectional study assessing the diagnostic accuracy of HCC detection in 440 patients with cirrhosis is ongoing (ClinicalTrials.gov Identifier: NCT03311152). Other promising single hypermethylation candidates include *VIM, FBLN1, TFPI2, TGR5, MT1M* and *MTIG*.[77], [78], [79]

Several studies have taken advantage of genome-wide methylome sequencing to identify combination methylation panels for improved detection of HCC.[80], [81], [82], [83], [84], [85] One study utilised methylated CpG tandem amplification and sequencing in a genome-wide detection of hypermethylated CpG islands in patients with HCC.^85^ In a small phase I pilot study involving 36 patients with HCC, 17 with cirrhosis and 38 healthy controls, the authors identified *RGS10, ST8SIA6, RUNX2* and *VIM* as high-performance markers for detection of small HCC (≤3 cm). The combination achieved a sensitivity of 94% at 89% specificity. Of interest, comparing DNA methylation between matched plasma and tissue samples from 10 patients with HCC, the authors found both cancer and non-cancerous tissues contributed to the elevation of the methylation markers found in the plasma of patients with HCC. In another larger study involving 1,098 patients with HCC and 835 healthy controls, the authors constructed a diagnostic panel of 10 methylated markers using methylation profiles of HCC tumours from The Cancer Genome Atlas in addition to an independent data set from normal blood leukocytes.^82^ When validated in cfDNA, the model achieved a sensitivity of 83% at 91% specificity (AUC 0.94) in distinguishing patients with HCC from normal healthy controls. Despite the excellent performance, a limitation is that controls were healthy individuals. A recent follow-up study involving 130 individuals (both patients with HCC and controls) reported similar results (sensitivity 95%, specificity 98%)^86^ leading to FDA breakthrough device designation (September 2019) for early detection of HCC. A clinical trial is ongoing to compare the performance of this panel alone, ultrasound alone or the combination of the methylation panel and ultrasound for the detection of HCC in patients with cirrhosis (ClinicalTrials.gov Identifier: NCT03694600). More recently, another study performed whole methylome discovery with identification of novel methylated DNA markers for HCC detection.^80^ Among 244 patients with HCC, cirrhosis or healthy controls, a 6-marker cfDNA methylation panel yielded similar sensitivity of 95% at 92% specificity (AUC 0.94). Importantly, the panel was able to detect 75% of patients with stage 0 and 93% with stage A HCC.^80^ In a follow-up study involving 136 patients with HCC and 401 controls,^87^ a panel of 3 methylated markers (*HOXA1, TSPYL5, B3GALT6*) in combination with sex and AFP showed 70% sensitivity and 89% specificity for detection of early-stage HCC. This panel has received FDA breakthrough device designation and further validation studies are ongoing (ClinicalTrials.gov Identifier: NCT03628651).

In summary, the aforementioned studies demonstrate that methylation profiling of plasma DNA has great potential for the detection of HCC; additional large prospective validation studies in cohorts of patients with cirrhosis undergoing active surveillance – who represent the ideal target population – are warranted.

### Analysis of mutations in ctDNA in HCC

The detection and analysis of somatic genetic alterations in ctDNA might be particularly useful in diagnosing disease, monitoring treatment response, and identifying mutations associated with treatment resistance. Additionally, ctDNA analysis may overcome the challenge of tumour tissue heterogeneity faced by focal tumour biopsy strategies. With improvements in NGS technology and a better understanding of the mutational landscape of HCC, several recent studies have performed a more comprehensive analysis of ctDNA with improved performance over that of single hotspot interrogation.^41^^,^^43^^,^^50^^,^^88^^,^^89^ In 1 study, exome sequencing showed that 83% of mutations identified in the liver were also detected in cfDNA.^89^ In another analysis of 30 HCC tissues and corresponding cfDNA using a targeted panel of 46 genes, ctDNA was detected in 63% of patients.^88^ The sensitivity of ctDNA increased to 87% in patients with large tumours (≥5 cm diameter) or metastatic disease. Not surprisingly, consistent with studies on cfDNA quantification and methylation, the ability to detect mutations in ctDNA is associated with tumour burden, vascular invasion and extrahepatic metastasis.^43^^,^^89^^,^^90^ Of interest, 81% of mutations detected in cfDNA were independently detected in the corresponding tumour biopsy.^88^ In agreement with other studies, the copy number profiles of ctDNA reflect the biology of the matched primary tumour.^33^^,^^43^^,^^59^

Several studies have shown that ctDNA mutation profiling can be used as a tool to monitor disease dynamics including response to treatment and disease progression.^38^^,^^59^^,^^89^ These studies showed that following resection, ctDNA levels dropped or disappeared completely and rose again prior to disease progression. A more recent study targeting 574 genes in tumour tissues of 3 patients with HCC revealed that 98%–99% of identified subclonal mutations were captured in ctDNA.^49^ Furthermore, the level of subclonal mutations changed in correlation with the patient’s tumour burden, with a lower fraction of mutated alleles detected after resection and a higher mutational frequency observed during recurrence. In a follow-up study of 34 patients with HCC, the authors performed WES to a median depth of 152x to identify specific single nucleotide variants (SNVs) and copy number variants (CNVs) in tumour tissue and peritumoral tissues.^91^ Leveraging individual findings, custom-made panels were designed to capture these mutations in patient’s plasma at a median depth of 7,204x. The threshold levels of SNVs and CNVs in ctDNA were detected in all preoperative patients with HCC and in 95% of patients at the time of tumour recurrence (compared to 49%, 45% and 77% for AFP, AFP-L3 and des-γ-carboxy-prothrombin [DCP], respectively). Serial measurement of ctDNA postoperatively identified 59% of patients with recurrence within 1 year, suggesting the feasibility of monitoring for minimal residual disease.

Furthermore, ctDNA carries genetic information integrated from the entire tumour mass, circumventing the challenges posed by intratumoural heterogeneity when only focal tumour biopsies are obtained.^59^^,^^92^ This concept was explored using shotgun massively parallel sequencing of plasma DNA in 4 patients with HCC before surgery and comparing it to multiregional sequencing of tumour tissue. The results revealed that up to 94% of tumour-associated SNVs were detected in cfDNA.^59^ Another study performed WES and targeted deep sequencing of 32 multiregional HCC tissue specimens from 5 patients, highlighting the challenges with mutation profiling of ctDNA.^92^ WES of ctDNA revealed only 18% of the mutations detected in tissue. When targeted deep sequencing was applied, detection increased to 84%. In this small study, the authors demonstrated that cfDNA captured most of mutations between tumour regions; however, this required a higher depth of sequencing to a median sequencing depth of 1,807x *vs.* 226x. Finally, a more recent study evaluating the concordance of ctDNA and tissue using a targeted panel of 8 genes among 51 patients with HCC found mutations in ctDNA from only 35% of patients.^93^ In patients with matched tissue DNA, 71% of mutations found in tissue were not detected in matched ctDNA. Thus, this approach lacked sensitivity.

A more exciting recent development in detecting HCC is the combination of DNA mutations with cancer-associated proteins. One study combining a panel of 4 genes (*TP53, CTNNB1, AXIN1, TERT*), AFP and DCP discriminated 65 patients with HCC from 70 without HCC, with 85% sensitivity at 93% specificity in the training cohort.^94^ The test yielded a sensitivity of 100% and specificity of 94% in the validation cohort of 331 at-risk patients; however, the positive predictive value was only 17%, reflecting the healthy younger training cohort in this study. In another study, Cohen and colleagues combined circulating proteins with NGS and detected early-stage cancer with sensitivities ranging from 69% to 98% for 5 cancer types.^95^ In liver cancer in particular, the assay achieved a sensitivity of 95% with over 99% specificity.

In summary, the variability in the proportion of patients with HCC and detectable ctDNA reflects not only cohort composition and methodologies of detection but also clinical characteristics of the disease. Nevertheless, these studies also showcase the feasibility and potential applicability of cfDNA as a diagnostic marker.

## Prognostic value of ctDNA in HCC

Outside of HCC detection, ctDNA can also play an important role in prognostication. Earlier studies showed shorter disease-free survival and overall survival were associated with higher cfDNA concentration,^52^^,^^65^ higher levels of cfDNA methylation markers,^39^^,^^40^ and specific hotspot mutations.^43^^,^^89^ In a recent study, analysis of 155 patients with HCC undergoing surgical resection showed that promoter methylation of insulin-like growth factor-binding protein 7 was associated with early tumour recurrence and decreased overall survival after hepatectomy.^96^ In 1 of the largest studies to date, including over 1,000 patients (Table 2), Xu and colleagues identified 8 methylation markers which independently predicted worse overall survival in both training and validation cohorts.^82^

**Table 2:** **Performance of ctDNA for disease monitoring or prognostication in selected studies**.

Study	Target	Patients	Performance	Limitations
**ctDNA mutation profiling**				
Kim *et al.*,^97^ 2020	2,924 SNVs in 69 genes	107 HCC	*MLH1* is associated with poor overall survival	Single *MLH1* target
Zhou *et al.*,^98^ 2020	1,021 gene panel (target not reported)	97 HCC, resected	Associated with shorter disease-free survival	Single liquid biopsy
Alunni-Fabbroni *et al.*,^90^ 2019	597 gene panel	13 HCC (SORAMIC trial)	cfDNA levels associated with presence of metastases and survival	Small sample size
Oh *et al.*,^99^ 2019	*VEGFA*, copy number alteration	151 HCC, 14 healthy controls	Higher cfDNA associated with shorter time to progression (sorafenib), shorter overall survival	Exploratory study
Cai *et al.*,^91^ 2019	CNVs and SNVs, AFP, AFP-L3, DCP	34 HCC, resected	High SNV and CNV correlated with shorter relapse-free survival and overall survival	Small sample size
**ctDNA methylation profiling**				
Xu *et al.,*^82^ 2017	8 markers	1,098 HCC, 835 healthy controls	Combined prognosis score predicted worse overall survival	Short follow-up

AFP, alpha fetoprotein; cfaDNA, cell-free DNA; CNV, copy number variants; ctDNA, circulating tumour DNA; DCP, des-γ-carboxy-prothrombin; HCC, hepatocellular carcinoma; SNV, single nucleotide variants.

Similarly, recent studies targeting specific mutations in cfDNA reflect intratumoral heterogeneity and predict poor prognosis. Kim and colleagues analysed 2,924 SNVs in 69 genes from 61 patients and validated SNVs in *MLH1, STK11, PTEN* and *CTNNB1* by digital droplet PCR.^97^ Of these, *MLH1* was found in both ctDNA and tumour tissue and was associated with shorter overall survival. In another study, analysis of >1,000 genes from 97 patients undergoing resection revealed that the presence of ctDNA 7 days after surgery was an independent predictor of poor prognosis.^98^ Twenty-one patients had at least 1 mutation and all of them recurred. A selection of recent studies have reported similar results for other genes (Table 2).^90^^,^^91^^,^^99^

## Current status and future considerations

**Fig. 2:**
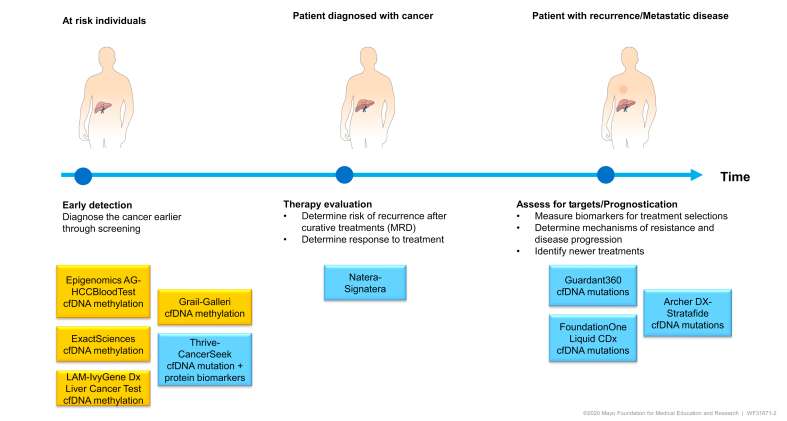
Clinical applications of cfDNA. Select examples of clinical tests having received breakthrough device designation or FDA approval. Colour denotes the technology used. cfDNA, cell-free DNA.

Precision oncology has undoubtedly transformed the clinical management of patients with cancer. With the improvements in NGS technologies accompanied by the increasing understanding of the molecular pathogenesis of HCC, ctDNA detection and characterisation carry immense potential for clinical application (Fig. 2). From early tumour detection to prognostication and therapy evaluation, several tests are in advanced stages of clinical development. In early tumour detection, 3 companies have received breakthrough device designation/CE Mark for their individual tests (ExactSciences,^100^ Laboratory for Advanced Medicine,^82^ and Epigenomics AG^75^). Another example includes the multi-methylation target panel, Galleri™ (GRAIL, Menlo Park CA). GRAIL launched the Circulating Cell-free Genome Atlas Study (CCGA), a prospective, observational, longitudinal clinical trial (NCT02889978) designed to determine detection and localisation of tumour origin in 50 cancer types by combining genome-wide cfDNA sequencing and machine learning. Recent updates from a sub-study cohort of >6,000 participants (>2,000 patients with cancer from >50 cancer types and >4,000 individuals without cancer) showed increasing sensitivity with disease stage (39% in stage I to 92% in stage IV), with tissue of origin localisation predicted in 96% of samples with 93% accuracy.^101^ GRAIL received breakthrough device designation in May 2019 and is planning to launch their product as a lab developed test in 2021. Another recent update included the first study of its kind where the authors combined the CancerSeek blood test (including 16 gene mutations and 9 protein biomarkers) with PET/CT to detect cancer in over 10,000 women without a history of cancer or symptoms.^102^ Twenty-six cancers were detected with sensitivity of 27%, specificity of 99% and positive predictive value of 19%. A new generation of this test has shown higher sensitivity without compromising specificity.^95^ This study showed the feasibility and safety of administering a cancer screening blood test with subsequent confirmation tests and imaging in a large prospective cohort. Only 38 women received false-positive test results and most of these women had non-invasive or minimally invasive testing. The design and application of this study illustrates the profound and near-term potential of cfDNA for the early detection of multiple cancers.

While the evaluation of cfDNA for early detection is a promising strategy, several outstanding issues warrant discussion. First, the lack of standardised protocols for preanalytical sample preparation and ctDNA purification, and different platforms for analysis, have resulted in significant variability in test sensitivity and specificity and hindered biomarker development in HCC. Standardisation of these factors would ensure consistency of results. The most promising approach, which is currently undergoing clinical validation, is DNA methylation profiling (Fig. 2). Previous work suggests that DNA methylation profiling is more broadly informative than mutation-based strategies which require WGS^103^ and targeted sequencing of much larger variants including CNVs and SNVs.^104^ Alterations in DNA methylation appear to be more prevalent than driver mutation sites and also provide an epigenetic memory of tissue of origin. They are therefore effective molecular markers for tumour detection.^101^ Exploiting combinations of DNA profiling with protein biomarkers have shown promising results as well and it is likely that multi-omics approaches will be developed for clinical use.

Second, while preclinical exploratory studies have generated significant proof-of-concept evidence of ctDNA as a surveillance blood test, large well-controlled longitudinal studies are still lacking. Of the 5 phases of biomarker development for early detection articulated by the Early Detection Research Network^105^ and more recently by the International Liver Cancer Association,^106^ there are few phase II clinical assay development studies and even fewer phase III studies that include independent prospective validation cohorts, particularly of patients with cirrhosis undergoing surveillance. Importantly, the detection of ctDNA mutations and methylations in advanced-stage cancer have been successful, however, current studies included low numbers of Barcelona Clinic liver cancer stage 0 patients (Table 1), which could lead to overestimation of biomarker performance for the early detection of liver cancer. Establishing acceptable sensitivity and specificity in this target population is crucial to avoid high rates of false negative/positive results. This lack of evidence and the off-target population are major impediments to biomarker development and clinical translation.^107^ Given the increasing interest and extensive research in identifying biomarkers for cancer detection and management, the International Liver Cancer Association also provided a framework on best practices in study design and interpretation of biomarker studies.^106^ The success of biomarker development will not only involve the right study design, including the target population, and interpretation but also investment in phase II studies and beyond by key players including government, industry and public-private partnerships.^108^ This evidence will be of paramount importance for the translation of these biomarkers into practice.

Finally, biomarkers must show clinical utility, demonstrating that they can improve health outcomes relative to the existing standard alternative and that the results can guide subsequent clinical management.^109^ The clinical utility of a diagnostic test is often measured as the expected number of life years gained with adjustment for the quality of those years.^109^ More evidence in the form of randomised clinical trials or decision analysis models of the clinical utility of cfDNA is needed. Importantly, there is substantial uncertainty in estimating the cost-effectiveness of these biomarkers in the detection of HCC.

In conclusion, the potential applicability of cfDNA as a biomarker for disease detection and prognostication in HCC has been clearly demonstrated. Its role in precision oncology is promising and will likely enhance cancer management for many patients in the near future.

## Financial support

LRR was supported by the Mayo Clinic Hepatobiliary SPORE (P50 CA210964) and the Mayo Clinic Center for Clinical and Translational Science (UL1 TR002377).

## Authors’ contributions

All authors contributed to the writing and review of this manuscript.

## Conflict of interest

Dr. Kisiel is an inventor of Mayo Clinic intellectual property licensed to Exact Sciences (Madison WI) and may receive royalties paid to Mayo Clinic under a sponsored research contract.

Please refer to the accompanying ICMJE disclosure forms for further details.
